# Mining Social Entrepreneurship Strategies Using Topic Modeling

**DOI:** 10.1371/journal.pone.0151342

**Published:** 2016-03-21

**Authors:** Yanto Chandra, Li Crystal Jiang, Cheng-Jun Wang

**Affiliations:** 1 Department of Public Policy, City University of Hong Kong, Hong Kong, China; 2 Department of Management, City University of Hong Kong, Hong Kong, China; 3 Department of Media and Communications, City University of Hong Kong, Hong Kong, China; 4 Computational Communication Collaboratory, School of Journalism and Communication, Nanjing University, Nanjing, China; 5 Web Mining Lab, Department of Media and Communication, City University of Hong Kong, Hong Kong, China; Utrecht University, NETHERLANDS

## Abstract

Despite the burgeoning research on social entrepreneurship (SE), SE strategies remain poorly understood. Drawing on extant research on the social activism and social change, empowerment and SE models, we explore, classify and validate the strategies used by 2,334 social entrepreneurs affiliated with the world’s largest SE support organization, Ashoka. The results of the topic modeling of the social entrepreneurs’ strategy profiles reveal that they employed a total of 39 change-making strategies that vary across *resources (material versus symbolic strategies)*, *specificity (general versus specific strategies)*, and *mode of participation (mass versus elite participation strategies)*; they also vary across fields of practice and time. Finally, we identify six meta-SE strategies―a reduction from the 39 strategies―and identify *four new meta-SE strategies* (i.e., *system reform*, *physical capital development*, *evidence-based practices*, *and prototyping)* that have been overlooked in prior SE research. Our findings extend and deepen the research into SE strategies and offer a comprehensive model of SE strategies that advances theory, practice and policy making.

## Introduction

The United Nations Millennium Development Goals (MDGs)—which range from halving extreme poverty and halting the spread of HIV/AIDS to providing universal primary education—have galvanized efforts from leaders and civil society organizations around the world to search for and develop better social intervention strategies. Although enormous progress has been made to date, unfinished tasks remain concerning the fulfillment of the Millennium Development Goals and the establishment of greater momentum to achieve the post-2015 agenda of a world of prosperity, equity, freedom, dignity and peace [1]. Among the most influential actors who have and will continue to play key roles in the attainment of the goals are social entrepreneurs: *entrepreneurial leaders who use the tools and logics commonly used by business entrepreneurs to achieve social goals*. Muhammad Yunus and his Grameen Bank’s collateral-free loans that alleviate poverty and the founder of APOPO (an acronym for Anti-Persoonsmijnen Ontmijnende Product Ontwikkeling: "Anti-Personnel Landmines Detection Product Development" in English) and his Hero Rats that detect landmines and tuberculosis are just two examples of a new movement that is engendering a sea change in the *way* society’s toughest problems are solved, people and institutions are shaped, and new goods and services are delivered to the public.

Despite the burgeoning literature on social entrepreneurship (SE) since the 1980s, very little research has examined the ways in which social entrepreneurs, as a global community-of-practice, create value to benefit the broader society. Importantly, there is a theoretical and empirical gap between theories on social entrepreneurship and what social entrepreneurs actually do, i.e., their *strategies* to solve society’s tough problems [2–5]. Our extensive review of the SE literature reveals that SE researchers have predominantly sought to explain social value creation from three perspectives: *organizational* [6–9], *motivational* [10,11], and *political* [12]. These studies complement the larger body of literature that attempts to define and achieve a *conceptual* breakthrough in the opaque field of SE [2,4,13,14]. While such research has significantly advanced our understanding of SE, it has largely overlooked the diverse approaches and strategies that social entrepreneurs have employed to achieve a social purpose. As Santos [3] notes, “the central unit of analysis for social entrepreneurs may be *the solution* and its underlying business model, *not the organization*” (p. 346). He notes that much can be gained by understanding that various models and strategies have been developed when the main driver is value creation. The primary argument here is that *solutions and strategies* are the most critical but least understood elements of SE. Although business strategy—the means to achieve outcomes—is a mature field in business management [15–17], we know very little about strategy in the context of SE, particularly what *social entrepreneurship strategy* means to self-identified social entrepreneurs. Moreover, prior research tends to be confined to small *n* studies and conceptual papers [9,18–21], which means that prior studies have focused on a small, static, and potentially restricted set of observations of reality. Although a few scholars have begun to study the phenomenon using larger datasets [22], these studies are quite limited, and little is yet known about what strategies social entrepreneurs employ to solve social problems.

A clear understanding of the nature and types of strategies employed by social entrepreneurs is critical for three primary reasons. First, it can help us model the diverse and widely used strategies of social entrepreneurs worldwide and to understand their dynamics over time. This work can spur research that tests hypotheses and assumptions to further advance SE theories and models. Second, research on SE strategies can offer useful insights for SE practitioners, social entrepreneurs and policy makers by identifying gaps and best practices. Finally, given the large range of strategies being studied, all of which are in published textual form, we can apply topic modeling―a new method from computer science and machine learning―to SE strategies, which can offer methodological advancements in SE, management and organizational research. This process is a methodological breakthrough in the SE field and in social science more broadly because it has been transferred from computer science and other scientific fields. To this end, we investigate two questions in this article: *What are the major social entrepreneurship strategies employed today*? *How have these strategies changed over time*?

This article contributes to SE strategy research in several ways. First, it synthesizes the social activism and change, empowerment, and SE streams of literature and identifies three key dimensions (resources: *material versus symbolic strategies;* specificity: *general versus specific strategies;* and mode of participation: *mass versus elite participation strategies*) as the foundation for understanding SE strategies. Second, using topic modeling, this article models and validates 39 distinct SE strategies that social entrepreneurs have used to pursue sustainable solutions to healthcare, environmental, social, political and economic problems along three key dimensions derived from the literature: *the nature of the resources used*, *the specificity of the strategy*, *and the mode of participation*. Third, using human coders, we reduce the 39 distinct SE strategies into six meta-strategies. In doing so, we offer *four new meta-SE strategies* that have not been identified in prior research. Finally, the study offers methodological innovation by showcasing the use of topic modeling to unpack and validate the diversity and dynamics of SE strategies associated with the practices of 2,334 social entrepreneurs supported by Ashoka, a nonprofit organization that selects and offers financial and technical support to the most impactful social entrepreneurs around the globe. In essence, this study opens up a new line of inquiry on SE strategies.

## Materials and Methods

### Ethics statement

This research was approved by the Human Subjects Ethics Sub-Committee of the Ethical Review Board of the City University of Hong Kong on June 16, 2015 (Reference Number: 2-2-201506_03). This Committee ensures that the research contained herein poses no threats or risks to human subjects. The Committee agrees that the data were analyzed anonymously and reported anonymously because this research focuses on aggregate patterns of strategies used by social entrepreneurs and not those used by specific social entrepreneurs. The data are available in the Dataverse: https://dataverse.harvard.edu/dataset.xhtml?persistentId=doi:10.7910/DVN/ATFL9A. The DOI number for the dataset is http://dx.doi.org/10.7910/DVN/ATFL9A. The R codes for the study are available at https://github.com/chengjun/datajournalism/tree/gh-pages/ashoka. The individuals described in this manuscript have given written informed consent (as outlined in the PLOS consent form) to publish their case details. Any questions or queries regarding the data and R codes can be directed to the corresponding author.

### Analytical approach

Before we conducted the data mining, we reviewed three streams of literature or models on how social leaders solve social problems: the social activism and change model [23,24] the empowerment model [3,25], and the resource- and capital-model of SE [22,26]. We used these models as an *initial* framework to synthesize the diverse SE strategies used over the past three decades. They encompass three dimensions: 1) the nature of the resources used; 2) the specificity of the strategies employed; and 3) empowerment practices. The nature of the resources used refers two types of resources―tangible/material (e.g., monetary assistance to start a micro business) and intangible/symbolic (e.g., creativity in education, awareness building on health issues). The specificity of the strategies employed refers to specific vs. general strategies or the degree to which an SE strategy is employed to address a specific social problem. For example, “healthcare treatment” is a specific strategy in healthcare, and “fair trade” is a specific strategy used in economic development; in contrast, general, broader strategies can be applied across two or more types of social problems such as “education,” which is a strategy that addresses poverty, healthcare, the environment, human rights and civic engagement problems. Empowerment practices can be viewed as both specific and general SE approaches; empowerment can be a specific strategy (e.g., equipping autistic individuals with computer programming skills) or a general strategy (e.g., developing self-efficacy and legal rights awareness for the low-income population, ex-felons, single mothers or immigrant groups).

There are at least two reasons behind the use of the three dimensions to test the emerging 39 strategies. First, there is general agreement that social entrepreneurs often face *“resource constraints”* [8, 26–28]. If a social entrepreneur does not have the resources to enact social change, how will he/she change the world? In other words, will he/she use more material or symbolic resources? Yunus’ Grameen Bank relies on *“changing the mindsets*, *lifestyle and habits of the poor”* through the *16 Principles of the Grameen Bank*, which every member needs to follow. However, anecdotal stories such as this and many others are insufficient to develop robust theory. The literature in entrepreneurship and social activism suggests that *symbolic action* is a very important strategy for entrepreneurs and activists [29–31] to acquire resources and achieve goals. However, the relative importance of symbolic and material strategies has yet to be tested in the social entrepreneurship literature.

Second, there is a need to deeply understand the types of strategies that social entrepreneurs *generally* use (generalizable across all fields of work) and those that are *field specific* (e.g., healthcare, human rights), and whether such strategies involve *high* (involving the masses) or *low participatory modes* (involving a few powerful elites). By identifying these dimensions of strategies and the content of the strategies (which will be demonstrated below), we will be able to enrich the *conceptual cornerstone* of social entrepreneurship behavior as well as provide contributions to the *definitional debate* on social entrepreneurship [13,14]. A clear conceptualization of general versus field-specific strategies, material versus symbolic strategies, and mass versus elite participatory strategies is also critical for practical purposes. For policy makers and social investors, such a categorization of strategies will allow them to better support social enterprises by focusing on the important strategies [27, 32]. For social entrepreneurs, this approach will help them invest in the relevant strategies to achieve higher performance.

One could, in principle, evaluate all SE strategies manually using a pencil and paper, but this evaluation process would fail when processing massive amounts of data from SE strategies. In the next section, we discuss the methods and data that we used to study the SE strategies of self-identified and selected and supported social entrepreneurs.

### Data: Strategy profiles of Ashoka Fellows

The legitimization and recent development of SE practices are largely due to a successful, worldwide SE support organization, Ashoka, which was founded in 1980 in Washington, D.C. to promote social entrepreneurship. Ashoka identifies and invests in leading or promising social entrepreneurs and helps them achieve maximum social impact. Ashoka also helps engage communities of entrepreneurs to promote social change and has created an infrastructure for SEs to access financing and business, academic and social change networks. Ashoka is much larger than the Schwab Foundation and Echoing Green, which reported approximately 300 and 600 fellows (past and present), respectively. Ashoka is the largest SE support organization worldwide and has more international fellows than any other such SE support organizations. It allows us to study social entrepreneurs using an extremely diverse database that spans geographic boundaries.

**Fig 1:**
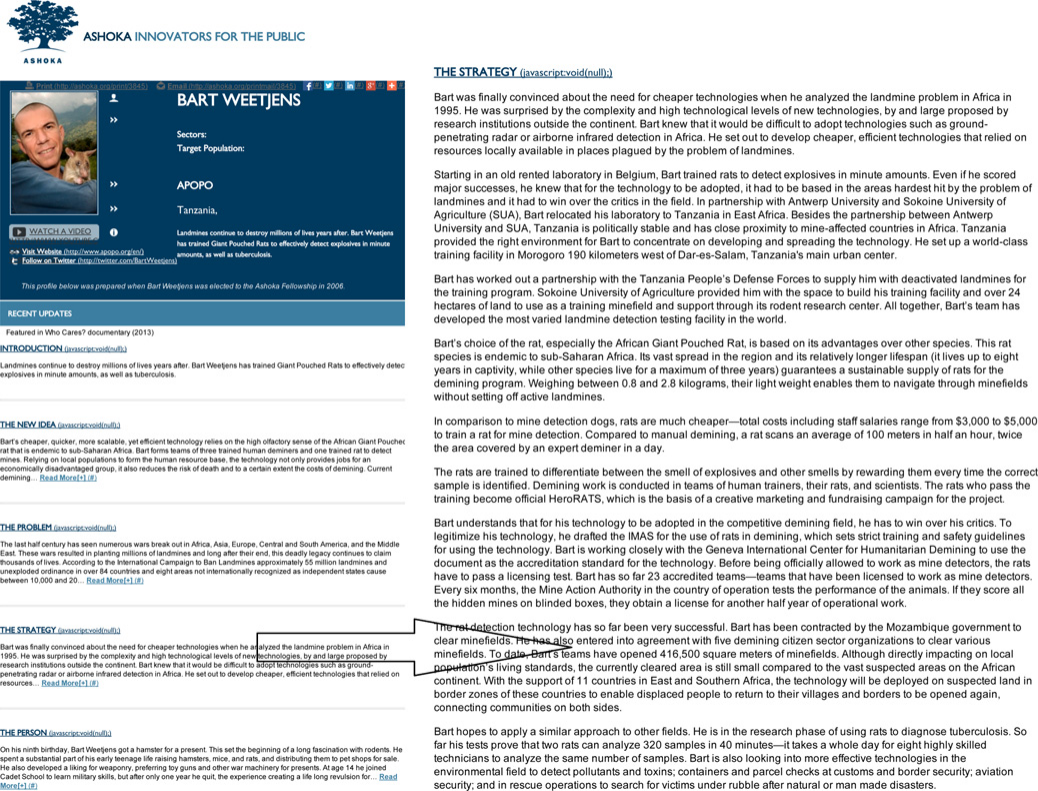
A sample screenshot of a profile page and the strategy profile of an Ashoka Fellow. Each sub-section in the Ashoka Profile (e.g., The Strategy) can be read fully by clicking the “Read More” link. A full display of the “strategy text” is shown on the right-hand side of the Ashoka Profile. Our analysis focuses on the “strategy text.”

To date, Ashoka has supported over 3,000 social entrepreneurs―the engines of social change in the 70+ countries in which Ashoka operates. Known as Ashoka Fellows, these social entrepreneurs are *among the world’s most powerful* agents for change, but they first must pass Ashoka’s strict selection process, which is anchored by five criteria: novelty of the idea or strategy, creativity, entrepreneurial quality, social impact of the idea or strategy, and ethical fiber of the social entrepreneurs (see Ashoka.org/support/criteria). Ashoka offers concrete *strategy profiles* (e.g., https://www.ashoka.org/fellow/jamila-abass) that describe the details of the SE strategies that have been used by its 3,000+ social entrepreneurs since 1982. Each strategy profile describes, in detail, the social entrepreneur’s strategy to achieve the desired social goals and is part of the public profile of each social entrepreneur supported by Ashoka (see Fig 1). Thus, Ashoka provides a natural laboratory to study SE strategies that cut across geographic boundaries, problem types, and time.

As noted earlier, our primary data of interest are the strategy profiles/texts of a pool of 2,334 Ashoka Fellows. Ashoka categorized the Fellows into six fields: civic engagement, economic development, environment, healthcare, human rights, and learning/education. Their profiles, which are available on Ashoka’s website [33], provide a comprehensive description of the SE projects, including organization and sectors, target populations, the focal problem, the strategy used to address the problem, an overview of the project’s activities, detailed descriptions of the innovative ideas and approaches, and the Fellows’ personal biographies (see Fig 1 for the biography and strategy profile of one Ashoka social entrepreneur). The individuals described in this manuscript have given written informed consent (as outlined in the PLOS consent form) to publish their case details. Our study focused on the texts that describe the 2,334 entrepreneurs’ strategies (hereafter “*strategy texts*”).

### Data preparation

**Fig 2:**
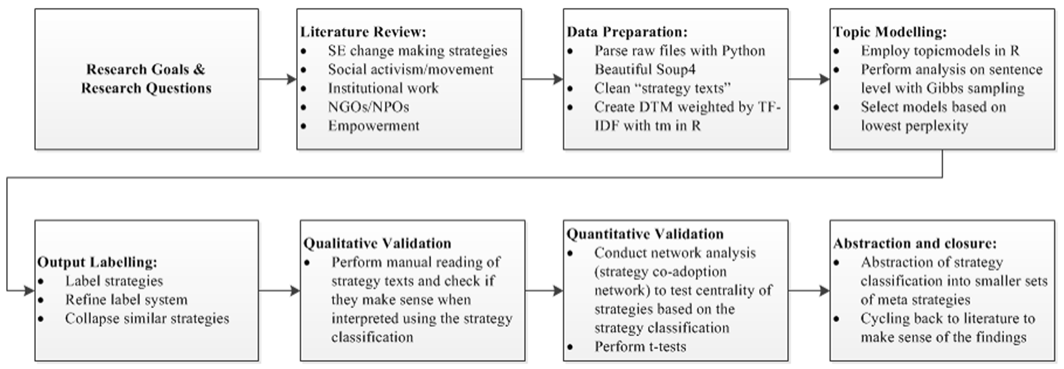
Design of the empirical study. DTM = document-term matrix; TF-IDF = term frequency–inverse document frequency; SPM = strategy person matrix; SYM = strategy-year matrix; tm = text mining package in R; Topicmodels = topic modeling package in R; igraph = network modeling package in R.

A total of 2,334 profiles of Ashoka Fellows were collected in July 2013. These HTML files were collected and pre-processed in several steps to create a profile corpus for text mining. First, we parsed the raw files using the BeautifulSoup package in Python, which is a library used to pull data out of HTML and XML files [34]. Second, we removed all HTML elements, stop words in English, numbers and punctuation characters and converted all of the text to lowercase. As mentioned previously, because we predominantly focused on the strategies used by social entrepreneurs, we decontextualized the strategy texts by removing the names of the entrepreneurs, their locations, and their organizations. Finally, using the *tm* package in R [35], we converted the strategy texts to a document-term matrix (DTM) to facilitate topic modeling. The DTM was weighted using term frequency–inverse document frequency (TF-IDF), whereby if we have a collection of N documents, the *frequency* of term t in document d is denoted as TF(t, d). Usually, TF(t, d) is calculated as the raw frequency of term t in document d, which is represented as f(t, d), or TF(t, d) = f(t, d). If the term t occurs in n_t_ of N documents, the inverse document frequency of term t is denoted as IDF = log(N/n_t_). Thus, TF-IDF is calculated as TF-IDF = TF*IDF [36]. For the sake of feature selection, only the terms with TF-IDF larger than the median were analyzed. Further details of the research design of this study are shown in Fig 2.

### Topic modeling using Latent Dirichlet allocation

We then developed topic models to identify social entrepreneurs' key strategies using the Latent Dirichlet Allocation model or LDA [37]. LDA is a generative model that infers unobserved meanings from a large set of observations. LDA is perhaps the most common topic model currently in use for automatic topic discovery; other topic models (e.g., correlated topic models) are generally extensions of LDA [38]. Topic models assume that each document contains a mixture of topics [39,40]. Topics are considered latent/unobserved variables that stand between the documents and terms. It is considered impossible to directly assess the relationships between topics and documents and between topics and terms. What can be directly observed is the distribution of terms over documents, which is known as the document term matrix (DTM). Topic models algorithmically identify the best set of latent variables (topics) that can best explain the observed distribution of terms in the documents. The DTM is further decomposed into two matrices—a term-topic matrix (TTM) and a topic-document matrix (TDM) [40]. The topics identified by the modeling are expressed probabilistically. Basically, certain terms and their associated documents are more likely to be associated with one topic than another. Each document can be assigned to a primary topic that demonstrates the highest topic-document probability and can then be linked to other topics with declining probabilities.

For example, assume K topics are in D documents, and each topic is denoted with Φ1:K. Each topic Φk is a distribution of fixed words in the given documents. Each document contains one or more topics. The topic proportion in the document is denoted as θd. For example, the *k*^th^ topic's proportion in document d is θd, k. Let w_d, n_ denote the n^th^ term in document d. Further, topic models assign topics to a document and its terms. For example, the topic assigned to document d is denoted as *z*_d_, and the topic assigned to the n^th^ term in document d is denoted as *z*_d,n_. According to Blei et al. [37], the joint distribution of *β*_1:k_,*θ*_1:D_, *z*_1:D_ and w_d, n_ plus the generative process for LDA can be expressed as:

$$p(\varphi_{1:\text{K}},\;\theta_{1:D},{\text{z}}_{1:D},\;w_{1:\text{D}})=\prod_{\text{i}=1}^{K}p(\varphi_{i})\prod_{d=1}^{D}p(\theta_{d})(\prod_{n=1}^{N}p(z_{d,n}|\theta_{d})p(w_{d,n}|\varphi_{1:K},z_{d,n})).$$ (1)

Note that*Φ*_1:k_,*θ*_1:D_, and *z*_1:D_ are latent, unobservable variables. Thus, the computational challenge of LDA is to compute the conditional distribution of *Φ*_1:k_,*θ*_1:D_ and *z*_1:D_ given the observable specific words in the documents w_d, n_. Accordingly, the posterior distribution of LDA can be expressed as:

$$p(\phi_{1:\text{K}},\;\theta_{1:D},{\text{z}}_{1:D}\;|\;w_{1:\text{D}})=\frac{p(\phi_{1:\text{K}},\;\theta_{1:D},{\text{z}}_{1:D},\;w_{1:\text{D}})}{p(w_{1:\text{D}})}\;.$$ (2)

Because the number of possible topic structures is exponentially large, it is impossible to compute the posterior of LDA [37,39]. However, topic models aim to develop efficient algorithms to approximate the posterior of LDA. There are two categories of algorithms: sampling-based algorithms and variational algorithms [39]. In our analysis, we primarily use the Gibbs sampling method, which is the most commonly used sampling-based algorithm in topic models. Using the Gibbs sampling method, we can build a Markov chain for the sequence of random variables (see Eq 1). The sampling algorithm is applied to the chain to sample from the limited distribution, and it approximates the posterior (see Eq 2) [39].

We employed the *topicmodels* package in R [41] to model the strategy texts. The tm package was also extensively employed for text preprocessing and converting the corpus to the document-term matrix [35]. In our analysis, we treated each sentence in the strategy text as a unique document because the strategy texts normally describe multiple strategies used by each entrepreneur. Following Blei et al.’s [37] guidelines, we specified a number of models with topic numbers ranging from 5–50 (with an interval of 5) and selected the model with the lowest perplexity as the final model. Perplexity is defined as

$$-\sum_{i=1}^{n}\frac{1}{n}\;{log}_{n}\;q\left(x_{i}\right)$$

where *q* is the model, *x*_*i*_ is the *i*^*th*^ document and *n* is the number of documents in the corpus [37].

Before we perform topic modelling analysis, we read a sample of 100 randomly selected strategy texts and agreed that the Fellows often used more than one strategy. Therefore, we decided to treat each sentence in the strategy text as a unique document and ran the analysis for 2,334 strategy profiles on the sentence level. We first performed the analysis on all of the strategy texts, and we then ran separate topic models for the six primary fields as defined by Ashoka (i.e., economic development, healthcare, civic engagement, environment, human rights, education). As mentioned above, the TTM assigned certain terms to each topic with higher probabilities. When the strategy topics mapping was finalized, we merged the data from the document attributes (e.g., the Ashoka Fellows’ names and the fellowship-award year) to create a strategy-person matrix (SPM) as well as a strategy-year matrix (SYM). SPM described the strategies co-adopted by the same person, while SYM reflected the strategies co-adopted within a particular year. The two matrices were later used to describe the adoption of strategies across entrepreneurs and over time.

## Results

We collected 2,334 strategy profiles for each Ashoka Fellow from 1982 to 2013. The distribution of the Ashoka Fellows across geographic regions and fields of practice, along with their years of appointment, is shown in Table 1. We also offer an interactive visualization that depicts the distribution of the Ashoka Fellows on a global map. The visualization is provided at http://computational-communication.com/globe/ashoka.html.

**Table 1:** Descriptive statistics of all Ashoka Fellows in the current study.

General description	# of Ashoka Fellows
Total number of collected postings	2334
***Length of posting on strategy***	
Average length	642
Minimum length	59
Maximum length	2044
Standard deviation of length	287
***Fellows per region***	
Asia	690 (29.6%)
South America	571 (24.4%)
North America	361 (15.4%)
Africa	322 (13.8%)
Europe	315 (13.5%)
MENA (Middle East and North Africa)	60 (2.6%)
Global	15 (0.7%)
***Field of work***	
Civic Engagement	422 (18.1%)
Economic Development	461 (19.7%)
Environment	282 (12.1%)
Healthcare	351 (15.1%)
Human Rights	450 (19.2%)
Leaning/Education	368 (15.8%)
***Year of joining Ashoka***	
1982**–**1990	164 (7.1%)
1991**–**2000	681 (29.1%)
2001**–**2010	1347 (57.7%)
2011**–**2013	142 (6.1%)

The number of new Fellows per year increased exponentially and reached its peak between 2001 and 2010; in addition, the distribution of fields of practice defined by Ashoka (e.g., the environment, healthcare, civic engagement, human rights, economic development, and education) is rather balanced. Asia and South America are home to the largest number of Fellows. The length of the strategy profiles ranges from 59 to 2,044 words (*M* = 642, *SD* = 287).

The topic modelling analysis in R *initially* generated 170 emergent categories of strategy topics across all of Ashoka’s six fields of practice (i.e., the Environment, Civic Engagement, Learning/Education, Human Rights, Health, and Economic Development fields). These six fields of practice were *pre-classified* by Ashoka in their selection and display of Fellows on their website. This pre-classification process is common among SE support organizations; for instance, Echoing Green pre-classified its Fellows into 8 fields of practice (i.e., Arts & Culture, Civil & Human Rights, Education, Environment, Food & Agriculture, Health & Healthcare, Poverty Alleviation & Economic Development, and Public Service & Civic Engagement; see http://www.echoinggreen.org/fellows), while the Schwab Foundation pre-classified its Fellows into 28 fields of practice that are similar to those of Ashoka and Echoing Green, albeit more “detailed” (e.g., Crime Prevention, Disabilities, Microfinance to Waste Management; see http://www.schwabfound.org/entrepreneurs). The initial 170 categories of strategy topics consist of the following: The Environment field generated 20 categories of strategies, while Civic Engagement, Learning/Education, Human Rights, Health, and Economic Development generated 30 categories each. For simplicity, we will call the categories of strategies “*topics”*. The categorization was performed based on the best perplexity measure for each of the fields, which are 1578.29 for the Environment, 1737.31 for Civic Engagement, 1460.23 for Learning/Education, 1657.82 for Human Rights, 1576.52 for Health, and 1739.52 for Economic Development (see the formula of perplexity in the Methods section above). Next, the three authors independently created labels for the 170 topics *manually* using a spreadsheet containing a matrix of the “terms” (or “words” that were automatically generated by the LDA algorithms) on the column side of the spreadsheet for the topic numbers (e.g., topic #1, #2,… #170) and on the row side of the spreadsheet for all of the topics. The LDA algorithms pulled together highly relevant “words” on a row so that when read horizontally, they reflect a topic; hence, different words on different rows reflect different potential “topics”. In the topic labelling process, each of three coders independently gave a *label* to each row of the *topic number* so that each topic has a *meaningful label* that best represents all of the terms that belong to it. As an illustration, taken from our dataset, the five important “words” *environment*, *conservation*, *awareness*, *education*, and *efforts* were labelled as “*enhancing environmental awareness*.” However, this LDA-based topic classification output and labelling process was only a *starting point* in our analysis; it was followed by a combination of *human interpretation* and *further quantitative validation*. This procedure followed Quinn et al. [42], who state that a *“topic model…requires user input after the initial quantitative analysis is completed…the user must spend more time interpreting and validating the results ex-post”* (p.225).

Next, due to the overlapping of many of the topic labels developed through the LDA outputs, the three authors discussed reducing the topic labels. After four rounds of intensive meetings, the authors reached a *consensus* to reduce the overall 170 topic labels to *70* in relation to each of Ashoka’s six fields of practice. The results of this reduction process are as follows: The Environment (7 topics), Civic Engagement (13 topics), Learning/Education (11 topics), Human Rights (11 topics), Health (15 topics), and Economic Development (13 topics). Based on these coding outputs, we noticed that there were still some overlaps among the topic labels in terms of their meaning. We subsequently *collapsed* topics with overlapping themes or content, resulting in a total of *39 topics* that cut across the Environment, Civic Engagement, Education, Human Rights, Health, and Economic Development. At this stage, we were satisfied that each of the topic labels was unique and non-overlapping. Our approach in combining human coding and automated text analysis followed similar suggestions made by various scholars (see [42–44]).

We list the 39 most common strategy topics, their distribution across all strategy profiles and the top five terms from each strategy. As shown in Table 2, a few strategies are dominant, as shown by their weight as a percentage of the total strategy topics identified using topic modeling. The top seven strategies involved the following: (1) engaging with vulnerable groups (13.9%); (2) training/educating (10.3%); (3) forming partnerships (9.5%); (4) engaging with the community (6.7%); (5) scaling up/replicating projects across the community/region (6.6%); (6) networking/sharing ideas and resources (5.1%); and (7) providing resource support (4.5%).

**Table 2:** The 39 strategy topics and top 5 words in each strategy.

Topics	%	Top 5 words
active participation	3.1	activity, participation, events, participate, meetings
awareness building	2.2	public, awareness, issues, media, attention
build facilities	1.2	services, center, based, created, together
collective action	0.8	issues, together, problems, needs, communities
**community engagement**	6.7	community, development, members, create, involved
community support	0.8	group, support, organization, efforts, among
conduct research	1.4	development, research, practices, international, project
diversified methods	1.4	many, different, approach, methodology, research
**engage vulnerable groups**	13.9	children, women, families, help, youth
fair trade	0.7	create, industry, trade, sustainable, fair
government support	1.5	government, funding, local, state, national
ICT	1.5	information, online, access, internet, tools
ICT/mobile therapy	0.6	therapy, mobile, used, develop, time
information-based practices	0.6	information, support, practices, another, providing
involve companies	0.8	project, companies, used, example, company
learning experiences	0.7	learning, experience, process, environment, classroom
legal enforcement	0.8	legal, cases, system, court, victims
life skills training	0.7	skills, learn, life, world, build
loans & financial support	1.5	bank, support, loans, provide, fund
marketing/distribution	0.7	products, market, markets, quality, marketing
media advocacy	1.6	radio, media, news, content, created
**networking/sharing**	5.1	network, organizations, ideas, share, practices
**partnership**	9.5	groups, community, organizations, network, partners
piloting & initiatives	2.2	first, project, program, open, began
policy making	3.6	government, public, policy, change, efforts
prevention	0.6	aids, drug, prevention, living, rehabilitation
protect vulnerable groups	0.8	people, disabled, young, disabilities, many
provide treatment	1.2	provide, healthcare, treatment, patients, cancer
public advocacy	0.8	national, international, level, policy, campaign
reform systems	1.3	education, system, health, ministry, create
religious leaders	0.8	community, members, youth, leaders, religious
**resource support**	4.5	support, provide, services, funding, resources
**scaling up/replication**	6.6	model, spread, plans, throughout, country
small business	0.7	business, small, businesses, entrepreneurs, company
sustainable practice	4.4	sustainable, forest, water, organic, farmers
train the trainers	1.4	training, teachers, teaching, programs, methods
**training/education**	10.3	training, program, education, students, school
use existing resources	1.4	resources, available, land, water, used
volunteering	1.4	volunteers, members, service, help, staff

We probed deeper and examined the importance or density of the 39 strategy topics across the six fields of practice using topic modeling. As Table 3 shows, each field of practice emphasizes different strategies. For instance, in economic development, the top three strategies are partnership formation (29%), training/education (8.35%) and the provision of loans and financial support (8.25%), while the environmental field’s top three strategies are creating sustainable practices (33.32%), community engagement (26.64%), and training/education (13.34%). For further details, see Table 3.

**Table 3:** The 39 strategy topics and the strategy topic density across fields.

	Civic Engagement	Economic Development	Environment	Health	Human Rights	Learning/ Education
active participation	**7.99%**	—	—	—	4.34%	3.69%
awareness building	—	—	6.70%	4.34%	4.36%	—
build facilities	—	—	—	**8.66%**	—	—
collective action	4.03%	—	—	—	—	—
community engagement	**8.02%**	4.15%	**26.64%**	0.00%	4.34%	3.70%
community support	—	—	—	—	4.35%	—
conduct research	—	—	—	4.35%	4.32%	—
diversified methods	—	—	—	—	—	**7.38%**
engage vulnerable groups	**12.00%**	**8.34%**	0.00%	**8.71%**	**21.69%**	**25.96%**
fair trade	—	4.17%	—	—	—	—
government support	—	—	—	—	4.35%	3.72%
ICT	4.01%	4.15%	—	—	—	—
ICT/mobile therapy	—	—	—	4.32%	—	—
information-based practices	—	—	—	4.33%	—	—
involve companies	3.98%	—	—	—	—	—
learning experiences	—	—	—	—	—	3.69%
legal enforcement	—	—	—	—	4.40%	—
life skills training	—	—	—	—	—	3.70%
loans & financial support	—	**8.28%**	—	—	—	—
marketing/distribution	—	4.14%	—	—	—	—
media advocacy	**7.99%**	—	—	—	—	—
networking/sharing	**11.98%**	4.16%	—	**8.67%**	—	3.68%
partnership	4.01%	**29.34%**	6.62%	8.63%	**8.68%**	—
piloting & initiatives	—	4.16%	—	—	4.33%	3.69%
policy making	4.01%	4.13%	**6.69%**	4.39%	—	3.71%
prevention	—	—	—	4.36%	—	—
protect vulnerable groups	—	—	—	—	4.39%	—
provide treatment	—	—	—	8.67%	—	—
public advocacy	—	—	—	—	4.37%	—
reform systems	—	—	—	4.40%	—	3.72%
religious leaders	—	—	—	—	4.35%	—
resource support	7.98%	—	—	4.34%	8.65%	3.72%
scaling up/replication	8.01%	4.13%	6.69%	4.39%	4.38%	**11.13%**
small business	—	4.21%	—	—	—	—
sustainable practice	—	4.14%	**33.32%**	—	—	—
train the trainers	—	—	—	—	—	**7.44%**
training/education	**12.01%**	**8.35%**	**13.34%**	**8.76%**	**8.71%**	**11.07%**
use existing resources	—	4.15%	—	4.34%	—	—
volunteering	4.00%	—	—	4.34%	—	—

**Fig 3:**
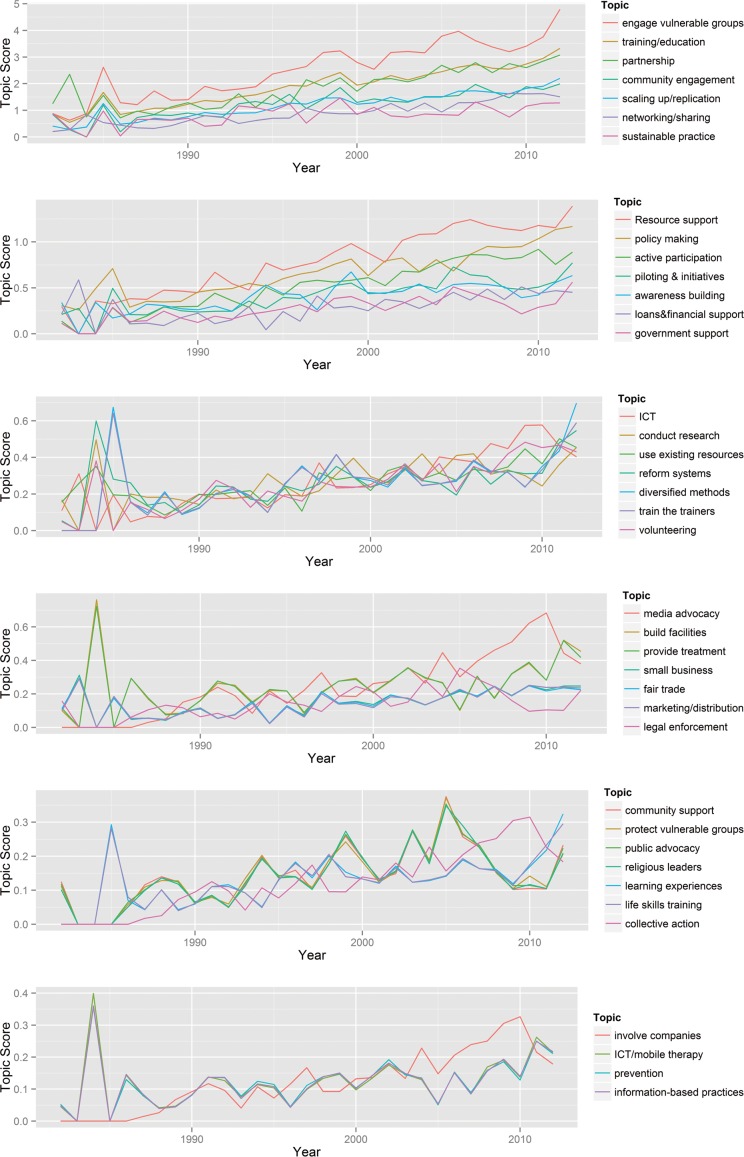
The dynamics of the 39 strategies (or strategy topics) over time (1982–2013).

To better understand the evolution of the importance of the different SE strategies over time, we plotted the *topic score* of each of the 39 strategy topics across 31 years, i.e., 1982 to 2013 (see Fig 3), using the raw score of all topics in the strategy-year matrix (SYM). The graphs demonstrate the relative importance and the increasing/decreasing trend for each of the 39 strategies, although the weight (or importance) of a few of the strategies remains close to what it was in 1982. Among the top strategies that show increasing trends are “engaging vulnerable groups,” “training/education”, “forming partnerships,” “community engagement” and “scaling up/replication of the social venture.” There were some spikes in the weight of certain strategies in the early years, such as an emphasis on “diversified methods” and “learning experiences” (important in the education field), “providing treatment,” “Information Communication Technology/mobile therapy in medical care,” and “information-based practices” (important in the healthcare field). The graphs also reveal the growing trend of “involving companies” in the project as a strategy (important in the economic development, healthcare, and education fields).

Drawing on the synthesis of the three strands of literature on social change above, we used three dimensions as a starting point to develop and validate a classification of social entrepreneurship strategies: 1) *the nature of the resources used* (material vs. symbolic focus); 2*) the specificity of the approach* (general vs. specific strategies); and 3) *the mode of empowerment/influence* (high participatory forms of action or mass influence vs. low participatory forms of action or elite influence). The three authors and three coders (i.e., two PhD students specializing in SE and a top undergraduate student who had recently completed a semester-long SE course) separately classified the 39 strategy topics produced from the earlier analysis along the three theoretical dimensions (i.e., material vs. symbolic strategies; general vs. specific strategies; and mass vs. elite participatory strategies). The two groups of coders coded the texts separately and then met and discussed their classifications. They were able to come to a consensus on the classifications at the end of the second meeting. The classifications by the two groups of coders (researchers and students) yielded very similar results, except for a few strategies that appear to be classifiable into more than one strategy category (e.g., prevention (healthcare), partnership, and government support). Specifically, the two groups of coders used a large whiteboard and drew a 2 x 2 x 2 matrix consisting of *Material* and *Symbolic* focus on the row side and *Mass* vs. *Elite Influence* under *General strategies* as well as *Mass* vs. *Elite Influence* under the *Field-specific strategies* on the column side. They then wrote down the 39 strategy topics one by one in the cells of the matrix and allowed each topic to be moved from one cell to another cell(s) as necessary. We started by asking, “is strategy topic X a material or symbolic strategy?”, and then we asked, “is it a mass or elite influence strategy?”; subsequently, we asked more precisely, “is it a general or field-specific strategy?” This process was performed iteratively until all of the topics were assigned; we allowed a topic to occupy more than one cell or category if the topic logically fit under two cells, which greatly enhanced the interpretation process and the assignment of strategy topics to relevant cells in the matrix. For instance, based on our manual reading of the strategy texts, “fair trade” practices always involve *material* (e.g., monetary or agriculture-related assistance) and *symbolic* (e.g., raising awareness of farmers’ rights) strategies, yet such practices are likely to be considered a form of *mass influence* (e.g., involving many farmers and rural communities) and a *field-specific* type of strategy commonly found in the *economic development* field. Thus, “fair trade” practices were classified in two separate cells (see the upper and lower third column of Table 4). Some strategies, such as *legal enforcement and engaging with religious leaders*, are essentially *symbolic*, rely on *elite influence* and are *field specific* in the *human rights* sector and were therefore classified in only one cell (see the bottom right cell of Table 4). The more *general strategies* (the four cells on the left in Table 4) are perhaps more interesting and important, as they cut across the fields of practice. We will use the classification of the 39 strategies along the 3 social change dimensions for the subsequent analysis and will validate them quantitatively (to be described next). The results of the human-based classification of the strategy topics along the three theoretical dimensions are presented in Table 4.

**Table 4:** A classification of validated social entrepreneurship strategies.

	General strategies		Specific strategies	
	Mass influence (direct)	Elite influence (indirect)	Mass influence (direct)	Elite influence (indirect)
**Material focus**	Community engagement, ICT/mobile therapy, Information-based practices, Build facilities	Partnership, Resource support, Government support	Sustainable practices (environment & econ), Fair trade (econ), Small business (econ), Loans & financial support (econ), Marketing/distribution (econ), Use existing resources (econ & health), Prevention (health), Provide treatment (healthcare)	Involve companies (civic), Marketing distribution (econ)
**Symbolic focus**	Community engagement, Training/education, Volunteering, Piloting & initiatives, Engage vulnerable groups, Active participation, Networking/sharing, Collective action, Scaling up/replication	Partnership, Policy making, Piloting & initiative, Media advocacy, Reform system, Government support, Scaling/replication	Awareness building (env), Sustainable practices (environment & econ), Fair trade (econ), Use existing resources (econ & healthcare), Life-skill training (edu), Learning experiences (edu), Public advocacy (human rights), Prevention (healthcare)	Diversified methods (edu), Train the trainers (edu), Legal enforcement (human rights), Conduct research (human rights), Religious leaders (human rights)

**Fig 4:**
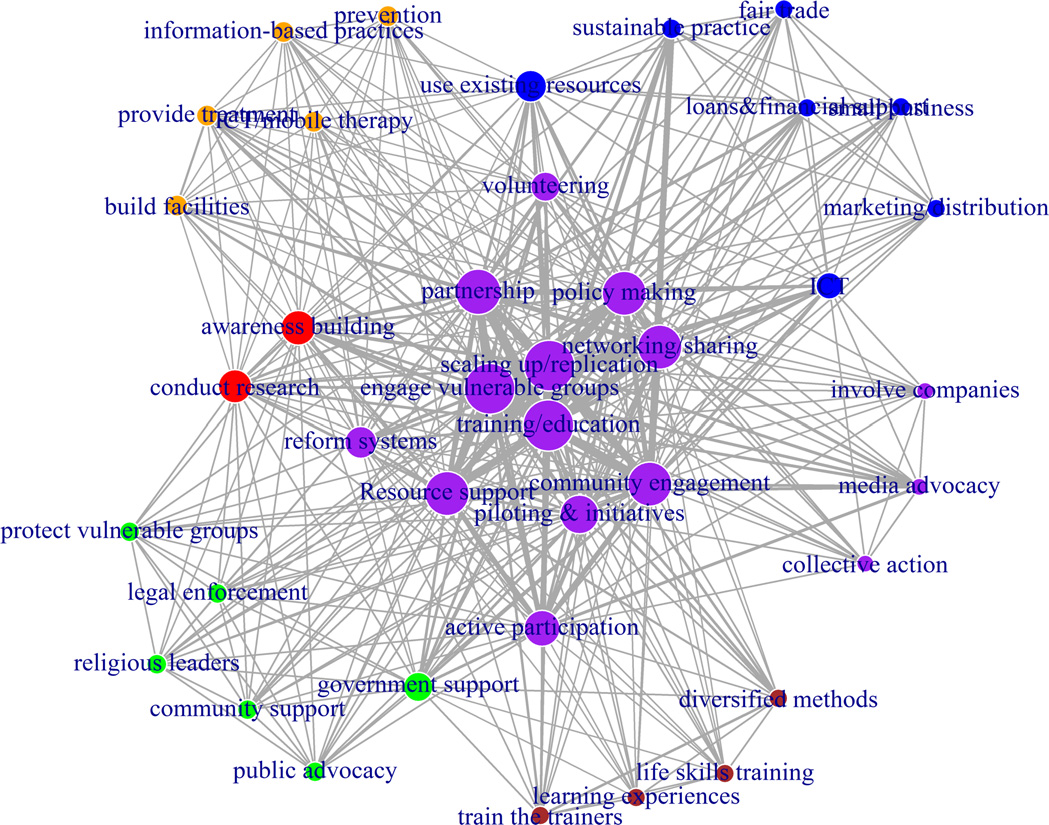
The co-adoption networks of the 39 social entrepreneurship strategies. The size of the vertex (node) is based on degree centrality (a larger node means a higher frequency of use of that strategy); strategies that are strongly co-adopted by Fellows have thicker edges (lines); and the colors represent different clusters of similar strategies. The network is plotted using the *igraph* package in R.

This strategy matrix was based on the strategies described in Tables 2 and 3 and Figs 3 and 4, which were re-classified using human coders and validated using network analysis.

Strategies may involve the use of resources with a *material* or *symbolic* focus or both. The former includes, for example, “building facilities,” “using ICT/mobile technologies,” “providing sustainable energy,” “providing loans and financial support,” and “providing treatment” (e.g., medicine), while the latter includes “training/education”, “engaging vulnerable groups,” “relying on volunteers,” “enhancing the importance of fairness through fair trade,” providing reasons for “sustainable practices” and “legal enforcement of specific issues.” The entire list of the material- and symbolic-focused strategies is shown in Table 4.

Some strategies denote a *general* type of solution that is applicable to more than one field (see “general strategies” in Table 4), while other strategies are *field-specific* (see “specific strategies” in Table 4). These general strategies include “engaging with vulnerable groups,” “forming partnerships,” “networking/sharing,” and “community engagement.” Some field-specific strategies include “sustainable practices” (environment), “diversified methods” and “train the trainers” (education), “loans & financial support,” “marketing/distribution” (economic development), “active participation” (civic engagement), “building facilities,” “providing treatment” (healthcare), and “legal enforcement” and “public advocacy” (human rights).

Strategies that emphasize empowerment through low participatory forms of action or *elite influence* rely on the power, resources and influence of key institutions in the society to enact change, such as engaging with companies, leaders in the government, and religious leaders, while “empowerment through *mass influence*/participation” strategies rely on more direct engagement with the masses to tackle social problems. As shown in Table 4, empowerment through elite influence includes the following strategies: “involving companies,” “forming partnerships with marketing experts,” “engaging experts to train the trainers,” “using leading research centers to provide objective views,” “engaging religious leaders to influence the public”, “partnering with government agencies and policy makers,” and “media advocacy.” The entire list of the elite and mass influence strategies is presented in Table 4.

### Validation of the social entrepreneurship strategy framework

#### The face validity of the 39 social entrepreneurship strategies

As a first step in ensuring the face validity of the classification of the 39 SE strategies, each of the three authors conducted a manual reading of 20 randomly selected Ashoka Fellows’ strategy profiles (60 non-overlapping profiles in total) to interpret whether the strategies from each profile fit with the classification. The results offered a good interpretation of the SE strategies within the framework. We provided two examples to depict a re-interpretation of the strategy profiles in relation to their classification. An Ashoka Fellow and the founder of APOPO (Anti-Personnel Landmines Detection Product Development, a Belgian social enterprise, www.ashoka.org/Fellow/), helps communities in Tanzania, Mozambique, Angola, Thailand and Cambodia detect landmines and tuberculosis using giant pouched rats. APOPO’s strategies include *building facilities* for giant rats (mass influence and material focus) and *training/educating the local people* (mass influence and symbolic focus) as well as healthcare-specific strategies such as *using existing local resources*, *conducting research* (mass influence and material focus and elite influence and symbolic focus, respectively) and *prevention* (mass influence and material focus). Another Ashoka Fellow, and the founder of Yayasan Prasasti Perdamaian (www.ashoka.org/Fellow/), offers culinary skills and stock ownership to help ex-terrorists reintegrate into society. His strategies include *building facilities* such as restaurants and bakeries, *training/educating* ex-terrorists (mass influence with material and symbolic focus), and *lobbying policy makers* and *media advocacy* (elite influence and symbolic focus). Specifically, he uses *small business* and *life-skills training* in culinary skills and running restaurants (mass influence with material and symbolic focus). In recent years, he also *engages companies* through partnerships with large firms (elite influence and material focus). This qualitative interpretation helps to ensure that the classification offers a logical categorization of the strategies and can be used to assist readers and the general public in understanding the strategies. An edited version of the strategy text showing the SE strategies used by one of the Ashoka Fellows who is the founder of APOPO (highlighted in brackets and shown in gray)—using manual reading—is shown below.

The founder of APOPO was finally convinced about the need for cheaper technologies when he analyzed the landmine problem in Africa in 1995….He set out to develop cheaper, more efficient technologies that *relied on locally available resources* (using existing local resources) in places plagued by the problem of landmines. Starting in an old rented laboratory in Belgium, he trained rats to detect explosives in minute amounts….In partnership with Antwerp University and Sokoine University of Agriculture (SUA), *he relocated his laboratory to Tanzania in East Africa* (building facilities). He *set up a world-class training facility in Morogoro* (building facilities) 190 kilometers west of Dar-es-Salaam, Tanzania's main urban center….He has forged a *partnership with the Tanzania People’s Defense Forces* (partnership) to supply him with deactivated landmines for the *training program* (training local people). Sokoine University of Agriculture provided him with the *space to build his training facility* (building facilities; partnership), over 24 hectares of land to use as a training minefield and support through its *rodent research center* (building facilities; conducting research). Taken together, his team has developed the *most varied landmine detection testing facility in the world* (building facilities). His choice of rat, particularly the African Giant Pouched Rat, is based on its advantages over other species. This *rat species is endemic to sub-Saharan Africa* (using existing local resources). Its vast spread in the region and its relatively long lifespan guarantees *a sustainable supply of rats* (using existing local resources) for the demining program. Weighing between 0.8 and 2.8 kilograms, their light weight enables them to navigate through minefields without setting off active landmines. In comparison to mine detection dogs, rats are much cheaper (using existing local resources)—total costs, including staff salaries, range from $3,000 to $5,000 to train a rat for mine detection. …The *rats are trained by local staff* (training local people) to differentiate between the smell of explosives and other smells by rewarding them every time the correct sample is identified. Demining work is conducted by teams of human trainers (training local people), their rats, and scientists. …He is working closely with the Geneva International Center for Humanitarian Demining (partnership) to use the document as the accreditation standard for the technology. Before being officially allowed to work as mine detectors, the rats have to pass a licensing test. The *rat detection technology* (prevention work) has so far been very successful. He has been contracted by the Mozambique government *to clear minefields* (prevention work). He hopes to apply a similar approach to other fields. He *is in the research phase of using rats to diagnose tuberculosis* (conducting research)….He is also looking into more effective technologies in the environmental field to *detect pollutants and toxins* (prevention work), *provide container and parcel checks at customs*, *assist in border and aviation security* (prevention work), and *engage in rescue operations to search for victims under rubble after natural or manmade disasters*.

Above and beyond the face validity check, we conducted further robustness tests to quantitatively validate the above framework, which we discuss in the next section.

#### General and specific social entrepreneurship strategies

Assuming that the 39 strategy topics were correctly categorized into the classifications described above (i.e., *material* vs. *symbolic focus; general* vs. *field-specific strategies*; *high participatory forms of action or mass influence* vs. *low participatory forms of action or elite influence*), we expected that our confidence in the classification would be manifested in the robustness test results. As discussed using the strategy profiles of the two Ashoka Fellows above, each Fellow typically uses two or more strategies, and a particular strategy can be adopted by multiple social entrepreneurs. *General strategies* tend to be more applicable across various social problems and fields of practice; thus, as one may expect, they should be “more popular” (more prevalent) than *field-specific strategies*. In other words, if the classification of the general strategies (vis-à-vis field-specific strategies) is correct, these general strategies should have been adopted by a greater number of Fellows than the field-specific strategies, thus rendering the general strategies *more central* and more widely used compared with the highly specialized field-specific strategies.

To test this prediction, we used a network analysis based on the logic of *strategy co-adoption* [45] to visualize the relationships between the strategies. The *igraph* package in R was used to construct and analyze the co-adoption network of strategies [46]. The *strategy co-adoption*, presented in the Strategy-Person Matrix (SPM), was defined as the frequency with which two strategies were adopted simultaneously by the same Fellow. If a Fellow uses two or more strategies, the nodes (which represent strategies) are connected by a line (see Fig 4), but if only one strategy is adopted by a Fellow, then such nodes will have no links. The more fellows there are who co-adopt strategies, the thicker the connecting line will be between the two strategies. Additionally, the more important a strategy is, the larger the node is (i.e., degree centrality; see [47]. We used community detection algorithms—the fast, “greedy” modularity optimization algorithm and the walktrap method—to identify local clusters of strategies [48,49]. In addition, we calculated and compared the average topic scores across general and field-specific strategies as another measure of the “popularity” of strategies.

We plotted the 39 strategy co-adoption networks by showing (1) the closely connected communities that emerged based on the strength of local connections and (2) the degree centrality (see Fig 4). The communities are highlighted by the node clusters with similar colors. The degree centrality is shown by the “size” of the nodes (larger nodes mean higher centrality). The edges or lines represent the strength of co-adoption (thicker lines between two strategies mean that the Fellows strongly co-adopted the two strategies). As shown in Fig 4, field-specific strategies tend to be located at network peripheries and have relatively smaller nodes (lower degree centrality), which imply a lower importance of such strategies compared with the general strategies that are located at the center and have larger nodes. These strategies include the “fair trade,” “loans & financial assistance,” “small business,” and “marketing and distribution” strategies from the Economic Development field, which are located at the periphery (northeast) of the network with lower centrality. The “prevention,” “information-based practices,” “provide treatment,” “ICT/mobile therapy,” and “build facilities” strategies from the healthcare field are also positioned at the periphery (northwest) of the network with low centrality. Specific strategies from the learning/education, human rights and civil engagement fields also appear at the periphery of the network and have small nodes.

**Fig 5:**
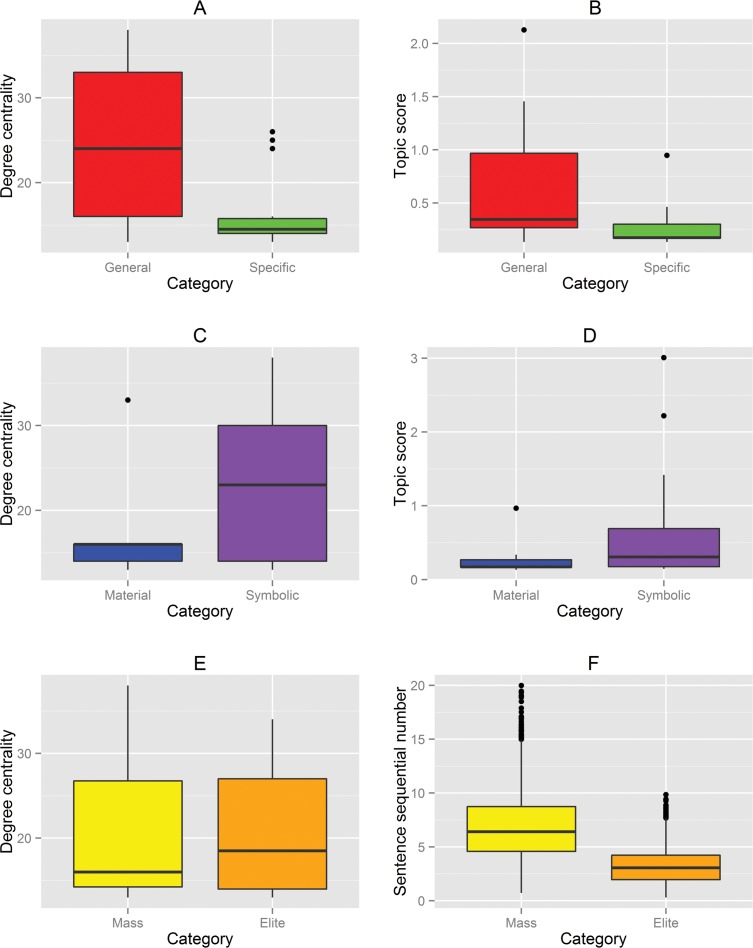
Robustness check of three dimensions of social entrepreneurship strategies. The box plots of the three dimensions of strategies—general versus specific, material versus symbolic, and mass versus elite participation—are shown based on their degree centrality and topic score.

Conversely, the general strategies are clustered in the center of the network and have larger nodes (higher degree centrality). This difference is statistically significant: general strategies have more links to other strategies than field-specific strategies, *t*(15.6) = 3.09, *p* < 0.001, and general strategies yield higher topic scores, *t*(13.6) = 2.29, *p* < 0.05 (see Fig 5, panels A and B, for boxplots of degree centrality and topic scores). Thus, general strategies are more important strategies and tend to be co-adopted by more Fellows. These general strategies include “training/education,” “scaling up/replication,” “partnership,” “engage with vulnerable groups,” “resource support,” “networking/sharing,” “community engagement,” “active participation,” and “policy making.” Thus, the network properties offer support to the above expectation that general strategies are used more often (i.e., are more popular) than field-specific strategies in the strategy co-adoption network of social entrepreneurs; thus, our classification clearly separates the general strategies from the specific strategies. In other words, in solving complex social problems, social entrepreneurs use multiple general and field-specific strategies.

**Fig 6:**
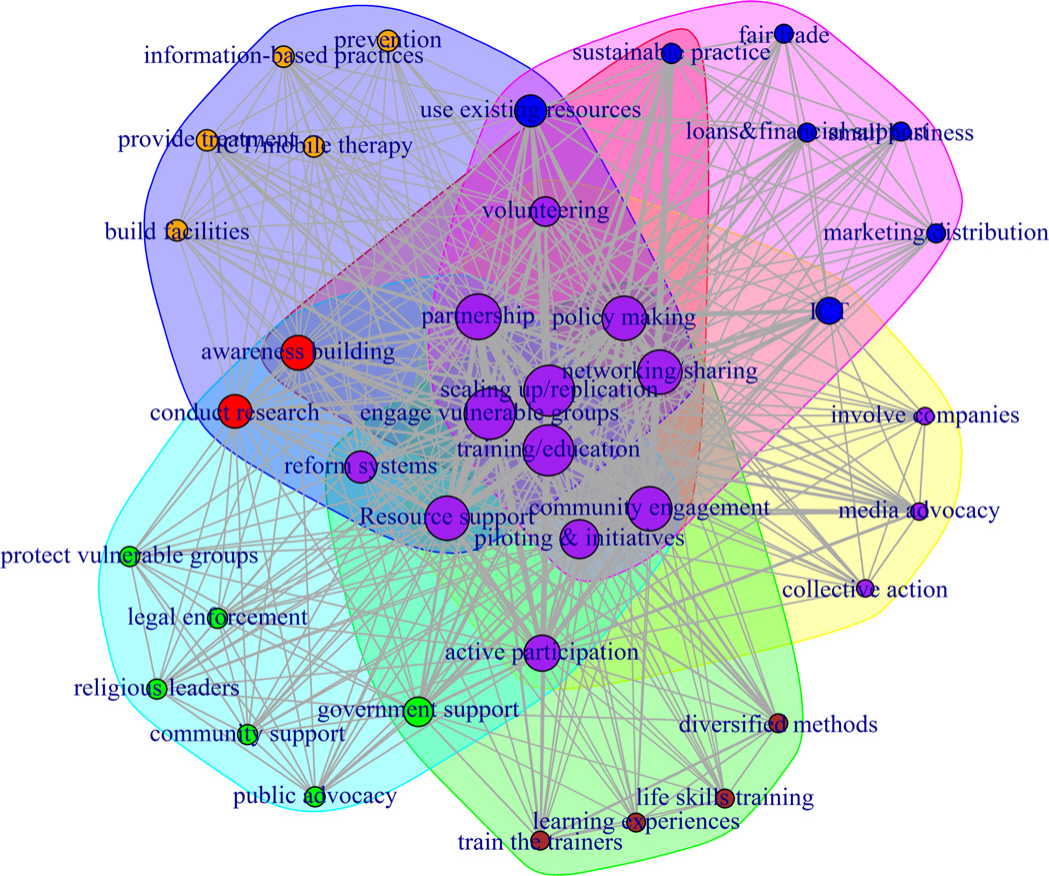
The clustering of the 39 social entrepreneurship strategies into the 6 fields of practice. The size of the vertex (node) is based on degree centrality. Strategies that are strongly co-adopted by a Fellow will have thicker edges (lines). The colors represent different clusters of similar strategies: there are six clusters of strategies in six unique node colors (blue, green, dark red, bright red, purple, yellow). The most central nodes are also in purple. The network is plotted using *igraph* in R.

Importantly, the network analysis of strategies in the classification shows strong “reproduction” of six distinct community clusters—which almost perfectly matches the six fields of practice used by Ashoka to classify the Ashoka Fellows. Had the strategies been misclassified, they would not have “loaded” or “clustered” to reproduce the six fields of practice. To better visualize how the 39 strategies are clustered into the six fields of practice, we repeated the network analysis and plotted another graph (see Fig 6) with the presence of each distinct practice field marked by uniquely colored clusters.

#### Symbolic and material social entrepreneurship strategies

When social entrepreneurs are said to create system-wide change, how and what exactly are they changing―the material or the symbolic world? In other words, which is the more influential strategy, the material or the symbolic strategy? The literature sheds some light on this question. Bornstein [50] notes that in creating systemic change, leading change makers focus on “shifting behavior patterns and perceptions” (p. 2), which implies the use of symbolic strategies. A closer look at two of the world’s most celebrated social entrepreneurs (and Noble Prize winners) Wangari Maathai, founder of the Green Belt Movement in Kenya [51,52], and Muhammad Yunus, founder of Grameen Bank in Bangladesh [53], reveals that women’s empowerment, environmental conservation and poverty eradication do not focus on money or tangible resources. Rather, the core aim of these strategies is to *change the public’s mindset*, *behaviors*, *lifestyles and perceptions*—the “symbolic” strategies—and the material strategies are expected to naturally follow. Grameen Bank’s success appears to rely on its micro-loans (a material strategy), but an in-depth look at the core of its strategy reveals the prominence of the Four Principles of Grameen―Discipline, Unity, Courage, and Hard Work―and the 16 Principles of “you shall and you shall not’ as the cornerstone of the mindset-, behavior-, and perception-changing strategies that are needed to alleviate poverty. The BRAC (Bangladesh Rural Advancement Committee), another highly successful micro-finance program that combats poverty in Bangladesh and across Asia, also employs the 17 Principles (e.g., we shall adopt family planning, we shall send our children to school, we shall try to be clean and always drink clean water). These principles are also “symbolic” strategies, without which the recipients of these micro-loans could end up using the proceeds for personal consumption or other personal gains, which would reduce the efficacy of Grameen’s and the BRAC’s poverty alleviation strategies.

Moreover, the literature notes that social entrepreneurs base their actions on quasi-universal principles or “orders of worth” to justify their beliefs, opinions and actions [22,54]: inspiration, the domestic world, fame, civic society, the market world and the industrial world. Of these principles, only the market and industrial worlds are “material” in nature; the rest are “symbolic.” Therefore, we expected that outstanding social entrepreneurs such as the Ashoka Fellows would focus on the symbolic world (the “why and how”) to a larger extent and that the material world strategy (the “what”) would naturally follow. A strong symbolic strategy can still work to achieve a social entrepreneur’s goals even with weak/little material resources because it can attract resources, investors and followers. However, the reverse may not hold true.

Therefore, if our findings on the classification of the symbolic strategies (vis-à-vis material strategies) are correct, the symbolic strategies should have been adopted by a greater number of Fellows, thus making these strategies more central, more widely used, and ultimately more popular than the material strategies. As the co-adoption network shows (Fig 4), the *symbolic strategies* tend to cluster in the middle of the network and thus have more linkages (higher degrees of centrality) than the *material* strategies, *t*(22.9) = 2.09, *p* < 0.05; furthermore, the t-test yielded marginally higher topic scores, *t*(26.1) = 1.86, *p* = 0.07 (see Fig 5, panels C and D, for boxplots of degree centrality and topic scores). This evidence suggests that social entrepreneurs tend to adopt more *symbolic strategies* than material strategies.

#### Elite and mass influence social entrepreneurship strategies

To test the prominence of low participatory forms of action or elite influence strategies vs. high participatory forms of action or mass influence strategies, we created a boxplot to visualize the degree of centrality of the two strategies. As shown in panel E of Fig 5, the degrees of centrality of the elite and mass influence strategies are not significantly different: *t*(22.8) = 0.0067, *p* = 0.946. This result suggests that both strategies are equally important on an aggregate basis when the time dimension is excluded. We also tested to see whether the use of elite vs. mass influence strategies relates to timing issues. The strategy text of the Ashoka Fellows describes each social entrepreneur’s strategies in chronological order, from the earliest to the most recent strategies used. Thus, we analyzed the strategy sentences chronologically and explored the relationship between the chronological numbering of sentences and mass/elite influence strategies identified in each sentence. The results, shown in panel F of Fig 5, indicate that elite-influenced strategies, on average, have a significantly smaller sequential number than mass-influenced strategies, *t*(3480.9) = 48.89, *p* < 0.001, which indicates that social entrepreneurs are more likely to adopt elite-influenced strategies in the earlier stages of their initiatives.

#### Toward a meta-social entrepreneurship strategy

To further refine our “initial SE strategy framework” into several primary concepts/constructs, the three authors independently coded the 39 strategy topics to obtain more general concepts. In doing so, two of the authors sat together and read each of the 39 strategy topics with the actual printed materials of the Fellows’ strategy texts at hand for reference. Then, strategy topics with similar meaning were collapsed manually until we did not see any clear overlap among the strategy topics. In performing the coding aggregation, we cycled among the 39 strategy topics and the relevant literature [55,56], thus transitioning from “inductive” to “abductive” research, where data and theory are considered together [57]. The third actor acted as a “devil’s advocate” to challenge and question the interpretation of each coding aggregation—as per Gioa et al. [55]. Thus, in essence, we started with an *initial* topic modeling analysis of 170 topics, gave labels to each of the 170 topics, *collapsed* them to 70 topics at the first stage of aggregation, finally reduced them to form the 39 strategy topics at the first-level coding and subsequently validated them qualitatively and quantitatively; finally, we used manual coding to identify more meaningful strategic themes grounded in the literature. After three iterations and discussions, we reached a consensus on six fundamental meta-SE strategies, as illustrated in Table 5.

**Table 5:** A meta-social entrepreneurship strategy.

Social entrepreneuring strategies (from topic modeling)	Meta social entrepreneurship strategies (using human coding)
training/education	Individual empowerment
provide treatment	
awareness building	
diversified methods	
learning experiences	
life skills training	
media advocacy	
prevention	
religious leaders	
protect vulnerable groups	
collective action	Collective action
community engagement	
active participation	
community support	
engage vulnerable groups	
involve companies	
networking/sharing	
partnership	
volunteering	
reform systems	Reform the system
policy making	
public advocacy	
government support	
legal enforcement	
train the trainers	
build facilities	Build physical capital
ICT	
ICT/mobile therapy	
loans & financial support	
marketing/distribution	
resource support	
scaling up/replication	
use existing resources	
small business	
fair trade	
sustainable practice	
conduct research	Evidence-based practices
information-based practices	
piloting/initiatives	Prototyping

Our six distilled SE strategies, shown in Table 5, are *individual empowerment*, *collective action*, *system reform*, *physical capital development*, *evidence-based practices*, and *prototyping*. *Individual empowerment*, *collective action*, and *physical capital development* appear to be the three most utilized grand strategies (associated with the highest number of strategy topics). These are followed by *system reform*, *evidence-based practices*, and *prototyping* as the next three most employed strategies. Of these, four SE strategies have not been explored or identified in prior SE research: *system reform*, *physical capital development*, *evidence-based practices*, *and prototyping*. Each of the six meta-strategies can be viewed as a tool to create social change at various levels—family, community, provincial, regional, national, and global levels. *Individual empowerment* aims to enhance individuals and communities (as target beneficiaries by, for example, using technical skills training and the influence of religious leaders to encourage and develop positive behavior and awareness-building to enhance individuals’ knowledge of their civil rights). *Collective action*, such as engaging with the community, local companies and volunteers, uses the power of collectives, which in turn strengthens individual and group empowerment. *Reforming the system* by exerting effort to change or enhance public awareness of powerful and resourceful actors/institutions can lead to positive change. For instance, community efforts to reform a system enhance individual empowerment because collective action brings people together to work effectively, which in turn, strengthens the “players’” sense of empowerment and can simultaneously improve the larger system or tackle a problem. *Building physical capital* enables actors to turn resource deficiencies into resource capacities—for instance, by using ICT/mobile technologies to deliver healthcare information to those deprived of medical access in rural areas, building schools and community centers to educate and help organize marginalized groups of people, and providing micro-loans to those denied access to conventional banking services. *Evidence-based practices* require using evidence and facts to examine social problems and devise solutions; they also serve as a mechanism to build the trust and integrity of a group, which in turn supports the group’s efforts to pursue a solution. *Prototyping* involves creating pilot schemes and models prior to launching a solution on a larger scale.

## Discussion

This article describes the findings from a data exploration and validation that spans 30 years of diverse SE strategies used by 2,334 social entrepreneurs affiliated with the global SE support organization Ashoka across 60 countries. Using three strands of social change literature—the social activism and change model, the empowerment model, and the resource- and capital-based model of SE—that describe how social leaders and entrepreneurs solve social problems as the study’s foundation, we distilled three initial dimensions of the nature of strategizing with a purpose as the raison d’etre of SE: *the nature of the resources used (i*.*e*., *tangible or symbolic)*,*the specificity of the approach (i*.*e*., *general or specific)*, and *the mode of participation (high participatory forms of action or mass influence or low participatory forms of action or elite influence)*. Our results first suggest that *social entrepreneurs rarely adopt a single strategy* but instead combine two or more strategies for their strategic plans. Second, the diverse strategies can be reduced into 39 SE strategies and fitted into a framework that depicts the *tangibility/symbolism*, *specificity/generality*, *and elite/mass participation* of the strategies (see Table 4 and Fig 4). We also show that the strategies vary in importance (i.e., density) across time and fields of practice (see Figs 3 and 6). Some of the most popular strategies in recent years include *building facilities*, *using ICT/mobile technologies*, *providing loans and financial support*, *encouraging training/education*, *engaging in partnerships with companies*, *promoting sustainable practices*, *enforcing legal remedies*, *engaging with religious and civic leaders for the cause*, and *advocating through the media* (see Fig 3). Our validation tests—using manual reading, network analysis of strategy co-adoption and t-tests—of the SE strategy types revealed the consistency and robustness of the classifications. Finally, we further reduced the 39 strategy topics into six types of SE strategies: *individual empowerment*, *collective action*, *system reform*, *physical capital development*, *evidence-based practices*, and *prototyping*. These represent the distilled categories of SE strategies.

Although our findings require further testing and exploration, they fill an empirical and theoretical gap regarding what social entrepreneurs actually do [2,4,5]. The SE research has focused on the organizational [6–9], motivational [10,11], political [12] and definitional [13,14] aspects of social entrepreneurship, but the diversity and key patterns in SE strategies are overlooked. We offer theoretically derived SE strategies associated with the social change, social activism, SE and empowerment literature and extend social entrepreneurship research. Specifically, the 39 SE strategies that we extracted can be mapped into three dimensions—using tangible or symbolic resources, general or specific strategies, and high participatory forms of action or mass influence vs. low participatory forms of action or elite influence. We then further classified these 39 strategies into six meta-SE strategies. Our findings extend and enrich prior exemplary studies on the various models of social activism and change [23,24,58], empowerment [3,25] and SE [22,26] by introducing *four* new and important *meta-SE strategies* that have been overlooked in prior research: *system reform*, *physical capital development*, *evidence-based practices*, *and prototyping*.

In the literature on social activism, particularly in the research on corporate social activism (i.e., activism against for-profit organizations), scholars [23] suggest that reformative activists are more likely to focus on reinstitutionalization than deinstitutionalization, to work with proactive firms, to challenge laggard firms and to mobilize more people (high participatory strategy), while radical activists focus on deinstitutionalization rather than reinstitutionalization and use material damage tactics. While their insights are interesting, Den Hond and De Bakker’s [23] piece is theoretical and lacks (large-scale) evidence. Bornstein [50] is among the first who uses the word “reform”–albeit as a passing reference–and provides an illustration of how a doctor named Vera Cordeiro “redefine[d] healthcare in Brazil” (p.130), where she changed the norm by providing care to poor children after they are discharged from public hospitals. The arguments above resonate well and converge with one of our four new meta-strategies called “*system reform*.” In essence, system reform is the core of what social activists do. When activists mobilize people, build coalitions with powerful elites, or use costly tactics (e.g., chaining themselves to railway tracks to get their voices heard), they seek to eventually reform the system. Our study provides empirical support with a large N to the presence of *system reform* as a strategy in social entrepreneurship, which has previously been reported in the social activism but not in the social entrepreneurship literature. What makes social entrepreneurship different from social activism, as in the example of Vera Cordeiro above [50] and the Ashoka Fellows who are the founders of APOPO and Yayasan Prasasti Perdamaian, is that social entrepreneurs create system reform differently from social activists. While social entrepreneurs rely on *innovation*, *trade and free will* to create social change, social activists (especially radical activists) largely rely on *non-trade* and *coercive power* to enact social change.

Zahra et al. [59] discuss the theoretical concept of “social engineers” who recognize “systemic problems with existing social structures and address them by introducing a revolutionary change” (p. 519). Zahra and colleagues [59] use the concept of “social engineers” as a process and outcome of SE, whereby social engineers are theorized to be creating a larger scale impact compared with the other two types in their typology (i.e., social bricoleurs and social constructionists). The *system reform* meta-strategy found in our study has some resemblance with Zahra and colleagues’ terminology of “social reform” (p.527). However, despite this resemblance, our strategy offers additional insights into the new concept of system reform, which consists of several dimensions (see Table 5) beyond Zahra’s generic “social reform”. More importantly, we offer empirical evidence using large-scale data of the existence of different types of system reform meta-strategies that consist of changing the social system/structure and the policies, conducting public advocacy, seeking government support, conducting legal enforcement and training the trainers. Therefore, our findings complement and extend Zahra et al.’s [59] research.

Mair, Batillana and Cardenas [22] suggest that social entrepreneurship models consist of four types of capital: political, human, economic and social capital. However, their study is limited to a relatively small sample of social entrepreneurs. Meyskens and colleagues’ [26] study on the resources and social value creation of 70 social entrepreneurs demonstrates the key resources used in social enterprises: organizations, financial capital, partnerships, innovativeness, and knowledge transferability. The social entrepreneurship strategies proposed by the scholars [22, 26] described above predominantly focus on the *sources of the resources* that social entrepreneurs use to create social change. Our new meta-strategy called *“physical capital development”* extends their research by suggesting that building physical capital is a common strategy to enact social change. For instance, in the case of the founder of APOPO, an Ashoka Fellow, developing a local infrastructure of *laboratories* to train African rats is a key element in his success performing landmine detection work. In the case of the founder of Yayasan Prasasti Perdamaian, another Ashoka Fellow, the development of *beefsteak restaurants* and other culinary businesses is central to his strategy of reintegrating former religious terrorists into society. In fact, the *scale and scope of the physical capital* is a good proxy to measure the success and social impact of these social entrepreneurs.

*“Evidence-based practices*” are a large part of what medical workers, psychologists, social workers, educators, and social policy analysts do in their daily work. Evidence-based practices, inspired by “evidence-based medicine,” refer to *the use of the best available evidence* to make decisions and solve problems [60]. For example, doctors and nurses rely on whatever actual evidence of cure is available in performing medical procedures or treatments. The use of evidence makes it easier for others to replicate and generalize such practices and allows for a trail of evidence that can be used to reveal the “truth” when malpractice occurs. Although evidence-based practices have their critics and supporters, such practices prevail and gain wide acceptance in social and organizational practices, including in business school education and in large companies [61]. In social entrepreneurship, there is a growing need to *measure and monitor the performance* of social enterprises as a way of enhancing their social and economic impact [27,62,63]. Such measurements are often required by funders so they can ascertain how well their money is spent and by social entrepreneurs to build their “theory of change,” i.e., how inputs translate into outputs. Thus, because social enterprises need to monitor their own performance, they inherently rely on evidence (at time t) to make better decisions (at time t+1). Prior research has not reported findings on *evidence-based practices* in social entrepreneurship. Our study offers empirical support with a large N for the importance of evidence-based practices as one of the new meta-strategies in social entrepreneurship.

Anecdotal stories show that social entrepreneurs experiment and *prototype* social solutions before they are scaled up to larger geographic regions or to the target beneficiaries [64]. Prototyping plays several roles in social entrepreneurship. First, it helps social entrepreneurs *reduce uncertainty* and provides *learning opportunities* to develop better intervention programs [28,65]. Second, it provides *signals* or *“evidence”* of the efficacy of a social enterprise’s solutions that in turn can be used to attract partners and funders [28]. However, these findings are based on small N studies. Our study offers a large-scale empirical validation of the importance of prototyping as a meta-strategy in social entrepreneurship.

Given that our strategies were derived from Ashoka Fellows’ strategy profiles, we offer a much more diverse “toolbox” of strategies than any previous SE study. By examining the local challenges and resource availability faced by each social entrepreneur along with their chosen strategies and strategic positioning, we can better understand the diversity of SE strategies and their effectiveness in certain situations. It is therefore important that we capture the diverse strategies used in our framework through the meta-strategies. To our knowledge, this study represents the first effort to systematically explore, classify, and validate SE strategies using a very large data set.

This article offers methodological innovation to research in the social entrepreneurship, strategic management, organizational science, and cognate fields. Specifically, it offers a novel approach (i.e., topic modeling) to extract latent dimensions of strategy data from a pool of data using machine-learning methods. In particular, it offers ways to efficiently analyze the dynamics and importance of strategy at a highly granular, temporal and field-specific level and examines how the strategies interact with one another. We demonstrate the efficacy of using topic modeling and network analysis as novel techniques to build and validate an SE taxonomy. Finally, the classification that we developed and validated in this study provides guidance to current and future social entrepreneurs regarding the strategies that have been successfully used by social entrepreneurs worldwide to effect impactful social change. It allows future change makers to replicate best practices from Ashoka’s social entrepreneurs and identify new social innovation opportunities, and it provides advice to policy makers and SE organizations, including nonprofits and NGOs, on how to better support social enterprises. Given the trends among NGOs to experiment with, spin off, or rebrand parts of or entire organizations according to the SE model, our findings offer insight into the range of SE strategies that they can adopt to achieve their goals. Our classification framework and meta-strategies also offer a “progress check” for policy makers and organizations such as the United Nations on workable SE strategies and ways to map the gaps to achieve certain national, regional and global social agendas. For Ashoka, as the “mother ship” of all Fellows, our findings can help the organization map strategies, pinpoint gaps, extend and/or tweak strategies and offer suggestions to further develop future Fellows. In the words of Drayton and Budinich [66], we are witnessing “a scale of [social] change not seen since the Industrial Revolution” (p. 58). This study has taken the first step in charting and understanding the actual strategies that thousands of elite change makers from 60 countries have employed over the past 30 years to help improve the human condition.

### Limitations and future research agenda

This study’s findings must be considered in light of its limitations. First, we did not study the differences between the strategies of successful versus less successful social entrepreneurs or those who were selected as Fellows or not. Our aim was not to identify predictors for such performance differences but to explore, classify, and validate the strategies used by social change agents selected by Ashoka. Future research may compare and contrast the strategies and SE environments of Fellows who failed versus those who succeeded in Ashoka, Echoing Green, Schwab and other SE support organizations. Second, this study focuses only on “strategy texts” but could be extended to other forms of corporate communication materials (e.g., websites, mission statements, recordings of meetings, blogs, and mobile conversations such as tweets and newspaper articles). We also assume that Ashoka has performed its due diligence to ensure the quality and validity of its Fellows’ strategy profiles described on its website. Third, our LDA model is sensitive to the values of the hyper-parameter of the Bayesian priors such that the results of the strategy dimensions extracted could be affected. We concur with Quinn and colleagues [42], who suggest that a “topic model requires more user input *after* the initial quantitative analysis is completed…users must spend more time interpreting and validating the results *ex-post*” (p. 225) and that LDA or other automated text analysis “is and should not replace other [human coding] method[s]” (p.225). The *sensitivity* of the hyper-parameter of the Bayesian priors in LDA could influence the results in terms of the *number of dimensions or topics extracted*. Therefore, based on our experience and reflecting the best practices developed by scholars before us [42–44], the LDA-generated topics are best served as *initial* outputs only, and they should be complemented using human interpreters via human-based coding and subsequent quantitative validation. LDA or other automated text mining methods and human coding can be used together to “powerful effect” that “build on and reinforce each other” [44] (p. 15–16). This aspect is perhaps the most critical point about using LDA for future scholars. We adhered to the best practices in performing the topic modeling analysis described above and acknowledged that the LDA approach has its own the strengths and limitations that can be complemented using other approaches, including human coding. Fourth, we did not examine social impact measures or the strategies’ impact on the specific goals that the social entrepreneurs aimed to solve. Fifth, we do not analyze the most frequent or infrequent words used in the word distributions of the strategy texts; doing so could offer additional insights from a linguistics perspective. Finally, future research can explore the uniqueness of each social enterprise’s strategies and compare the strategies across social enterprises. These limitations, if explored, could offer rich avenues for further research.
